# Microbial genome (Illumina MiSeq) sequencing of drinking water treatment residuals to evaluate compatibility with environmental applications

**DOI:** 10.1007/s10661-023-11511-3

**Published:** 2023-08-09

**Authors:** Tomi Turner, Daniel Tonge, Helen C. Glanville, Rebecca Wheeler, Ian W. Oliver

**Affiliations:** 1 School of Geography, Geology and the Environment, Keele University, Keele, ST5 5BG UK; 2 School of Life Sciences, Keele University, Keele, ST5 5BG UK; 3 4R Group, Control House, A1 Business Park, Knottingley Road, Knottingley, WF11 0BU UK; 4 School of Social Sciences and Humanities, Loughborough University, LE11 3TU Loughborough, UK

**Keywords:** 16S rRNA, Genomics, Water treatment residual, Canonical correspondence analysis, Species richness, Microbial diversity

## Abstract

The clarification of drinking water leads to the production of large quantities of water treatment residuals (WTRs). DNA was extracted from six WTR samples collected from water treatment plants within the UK to compare their bacterial communities and examine whether factors such as coagulant usage (aluminium versus iron salt), the type of water source (reservoir or river), or leachable chemical composition influence these communities. Bacterial 16S variable region 4 (V4) was amplified and sequenced using Illumina MiSeq sequencing. The most abundant phyla in WTR samples were *Proteobacteria*, *Actinobacteria*, *Bacteroidetes*, *Acidobacteria*, and *Firmicutes*, collectively representing 92.77–97.8% of the total bacterial sequences. Statistical analysis of microbial profiles indicated that water source played a significant role in microbial community structure, diversity, and richness, however coagulant type did not. PERMANOVA analysis showed that no single chemical variable (pH, organic matter, or extractable element concentration) influenced microbial composition significantly; however, canonical correspondence analysis of WTR microbiomes yielded a model using all these variables that could be used to explain variations in microbial community structures of WTRs (*p* < 0.05). No common, potentially toxic cyanobacteria, or related pathogens of concern were found. Analysis with PICRUSt showed that WTRs all had similar predicted microbial functional profiles. Overall, the results indicate that WTRs analysed in this study are unlikely to pose any threat to soil microbial community structure when applied to land as a soil conditioner or enhancer and may help to enhance the soil microbial community.

**Supplementary Information:**

The online version contains supplementary material available at 10.1007/s10661-023-11511-3.

## Introduction

During the treatment of drinking water, suspended sediment and other impurities are typically removed by a process of coagulation and flocculation brought about by dosing with aluminium or iron-based salts. This produces clean water fit for human consumption but also generates large quantities of water treatment residuals (WTRs) as the by-product (estimated to be up to 1–3% v/v of treated water), comprising the aluminium or iron salt derivatives and any removed impurities (Dassanayake et al., 2015). Once dried WTRs are in some ways comparable to soils as they consist of iron and aluminium oxi-hydroxides, organic matter, and varying amounts of other elements including many that are important plant nutrients such as nitrogen, copper, nickel, and zinc (Turner et al., 2019). Therefore, in an effort to move towards a more circular economy and the sustainability ideal of recycling valuable nutrients, and also to avoid or reduce the escalating costs of disposal via landfill or incineration, WTRs are increasingly being applied to land for general soil improvement and nutrient additions (Turner et al., 2019). It has been determined that the main contributors to the chemical properties of WTRs are the properties and characteristics of the raw water source and the type of coagulant used during treatment (Al or Fe) (Babatunde & Zhao, 2007; Turner et al., 2019). There has however been very little research conducted to date on the microbial suites within WTRs or the potential influence that additions of WTRs might have on native soil microbial communities; hence, the microbial community composition of WTRs and any influence it might have on soil microflora are still poorly understood. The overall abundances of culturable anaerobic microbes in WTRs have been reported to be similar to those found in soils (Oliver et al., 2011), and in one study the application of Fe-WTRs to soils was found to increase the total culturable heterotrophic bacteria within soils (Garau et al., 2019). Studies analysing WTR treated soils (aged between 5 days and 6 months) employing gradient gel electrophoresis staining and Biolog EcoPlates (Biolog Inc., Hayward, CA) have found that the land application of WTRs at up to 25% (w/w) led to no negative impacts in terms of microbial biomass or total heterotrophic bacteria, fungi, and actinomycetes (Garau et al., 2014; Pecku et al., 2006).

More recently, a small number of studies have emerged that extracted and sequenced the DNA from WTRs (Wang et al., 2021a, b; Xu et al., 2018). For example, Xu et al. (2018) analysed six WTRs from China and determined that the principal phyla were *Proteobacteria*, *Bacteroidetes*, *Firmicutes*, *Verrucomicrobia*, *Planctomycetes*, and, notably, *Cyanobacteria*. The results highlighted the presence of potentially toxic cyanobacteria (*Planktothrix*, *Microcystis*, and *Cylindrospermopsis*) and some potential pathogens (e.g. *Escherichia coli*). Ai et al. (2020) analysed WTRs produced from water sources in the USA affected by cyanobacterial blooms and found that, despite being continents apart, the dominant phyla were similar to the above study and comprised *Proteobacteria*, *Bacteroidetes*, *Firmicutes*, *Actinobacteria*, and again *Cyanobacteria.* Therefore, the question remains as to whether WTRs in other regions have similar bacterial profiles and whether any micro-organisms of concern are prevalent in WTRs generally. The present study aimed to address this question by analysing the bacterial compositions of WTRs sourced from six treatment plants across the UK (from England, Scotland, and Wales) spanning a variety of water source types (river or reservoir), principal treatment salts (aluminium or iron), and chemical compositions, enabling an assessment of the influence of these variables on bacterial community composition.

## Materials and methods

### Sample collection and preparation

#### Water treatment residual samples

Samples of WTRs were obtained from the storage tanks and lagoons of six water treatment plants located within the UK (England, Wales, and Scotland) and stored in 1-L HDPE bottles. The individual plants were selected to cover a range of geographic locations, principal coagulant used (i.e. aluminium or iron salt), and raw water source type (i.e. river or reservoir) (Table 1). At the time of collection, these WTRs had been stored for a period that was typical for each plant before being transported off site (in many cases for land spreading). WTRs can be removed from a water treatment plant daily, or they may be stored for months before being removed. This storage time varies by site but also depends on the season and demand for WTRs. This makes it difficult to estimate an exact storage time for each site. WTR properties have been found to change with dewatering (Wang et al., 2021b). Therefore, because of WTR differing levels of dewatering at the different plants, the collected WTRs were dewatered to a more consistent level by being centrifuged at 855 RCF (Relative Centrifugal Force) for 10 min and the supernatant removed via syringe. The remaining sediment was stored at − 20 °C until further use.

**Table 1:** Mean (*n* = 3) 0.01 M CaCl_2_ extractable element concentrations (bioavailable fraction), organic matter content, and pH (1:5 de-ionised water) of WTR samples

Sample	WTR 1	WTR 2	WTR 3	WTR 4	WTR 5	WTR 6
Coagulant type	Fe	Al	Fe	Fe	Al	Al
Source	Reservoir	River	River	Reservoir	Reservoir	Reservoir
Country of origin	Wales	Wales	England	Wales	Scotland	Scotland
Al (mg/kg)	2.08 ± 0.043	1.87 ± 0.086	1.54 ± 0.00049	0.289^*^	1.30 ± 0.14	3.40 ± 0.048
Cu (mg/kg)	0.316 ± 0.033	0.424 ± 0.013	0.601 ± 0.026	0.0455^*^	0.0592 ± 0.0026	0.0334 ± 0.017
Fe (mg/kg)	70.9 ± 3.5	bdl	bdl	0.108^*^	1.51 ± 0.15	10.9 ± 0.30
K (mg/kg)	26.5 ± 0.61	48.2 ± 2.3	47.9 ± 0.34	14.3^*^	259 ± 18	35.1 ± 2.0
Mg (mg/kg)	25.2 ± 0.88	67.5 ± 3.1	112 ± 1.5	73.1^*^	71.5 ± 5.2	33.2 ± 2.7
Mn (mg/kg)	13.3 ± 0.76	38.7 ± 0.78	8.80 ± 0.23	7.71^*^	25.7 ± 1.9	58.9 ± 3.9
Na (mg/kg)	173 ± 5.2	274 ± 9.5	169 ± 0.82	59.4^*^	46.9 ± 4.3	34.5 ± 2.8
pH	7.91 ± 0.24	7.26 ± 0.30	7.71 ± 0.18	7.85 ± 0.20	6.89 ± 0.16	7.25 ± 0.13
Organic matter content	33.2 ± 0.8	38.5 ± 1.2	19.1 ± 0.6	27.2 ± 0.3	35.8 ± 0.2	44.2 ± 0.8

*bdl*  below detection limit

^*^Single replicate only analysed for extractable elements due to limited sample quantity

## Organic matter, pH, and extractable element concentration determination of WTR samples

The pH of samples was determined using a handheld Hannah pH meter in a 1:5 solid:de-ionised (Milli-Q) water mixture. Organic matter content was determined by loss on ignition; samples were dried at 105 °C for 24 h before placing 5 g subsamples (*n* = 3) in a muffle furnace at 550 °C for 4 h. The bioavailable fraction of elements in WTRs was estimated through batch extraction experiments using 0.01 M CaCl_2_ (Houba et al., 2000; Houben et al., 2013; Oliver et al., 2005). To accomplish this, subsamples of WTRs were dried at 105 °C for 24 h before taking 2 g subsamples (*n* = 3) of each and adding 20 ml of 0.01 M CaCl_2_ solution. Samples were equilibrated in an end over end shaker for 2 h, centrifuged for 5 min at 855 RCF (Relative Centrifugal Force), with the supernatants then collected, filtered (0.45-µm syringe filter), acidified with 100-µl high purity HCl, and stored at 4 °C until analysis by ICP-OES (Optima 5300 DV, Perkin Elmer, UK). A summary of the physical and chemical properties of WTRs is presented in Table 1.

## DNA extraction

The extraction of DNA from substrates was conducted using the Qiagen DNeasy PowerSoil Kit (Qiagen, Hilden, Germany) according to the manufacturer’s protocols. DNA was extracted from 0.25 g subsamples of each dewatered WTR. DNA was extracted in replicate samples of each WTR (*n* = 3 generally and *n* = 2 for WTR 2) to assess homogeneity of samples. Extracted DNA was stored at 4 °C until downstream use. All plasticware and extraction solutions were exposed to UV light prior to conducting the DNA extractions to reduce the risk of contamination from plasticware or the laboratory environment. Further, a control sample negative blank comprising molecular grade water (RNase-, DNase-, and protease-free) was processed through the entire DNA extraction procedure in parallel to control for this eventuality.

## PCR amplification and sequencing

Extracted DNA was subjected to nanodrop analysis to check the purity and concentration of extracted DNA. Following this quality assurance protocol, subsamples of extracted DNA were analysed by Eurofins Genomics where the V4 region of bacterial 16S rRNA was amplified and Illumina MiSeq sequencing was conducted (Bisht et al., 2018).

## Bioinformatic analysis

Bioinformatic analysis was performed using QIIME implemented as part of the Nephele 16S paired-end QIIME pipeline using open reference clustering against the SILVA database for bacteria at a sequence identity of 99%. All other parameters remained as default (Weber et al., 2017). This process resulted in an average 63,424 reads for WTRs.

Tags were clustered into operational taxonomic units (OTUs) which were assigned based on their sequence similarity to the SILVA 99 (v132) database. Analysis of core diversity was conducted considering the best taxonomic classification in each instance. Down sampling was not used due to the inclusion of a negative blank.

The genomic functional profile of samples was determined using PICRUSt (phylogenetic investigation of communities by reconstruction of unobserved states) (Douglas et al., 2018). This method uses marker genes and the Kyoto Encyclopaedia of Genes and Genomes (KEGG) reference genome databases (www.genome.jp/kegg/) to predict the metagenome functional content of samples without the need for metagenomic analysis (Kanehisa et al., 2016).

## Statistical analysis

Bacterial community data was square root transformed to reduce the potentially distorting influence of large counts of certain genera (Lenehan et al., 2017); all further analysis discussed were conducted on this transformed data. The statistical significance of differences between samples in terms of taxonomic richness and diversity of genera (quantified via the Shannon diversity index; Eq. 1) were explored using ANOVAs in R following appropriate checks for adherence to normality and associated underlying assumptions.

1 $$\begin{array}{c}Shannon\;index=-\sum\limits_{i=1}^{s}{p}_{i}\;ln\;{ p}_{i}\end{array}$$

where *p* is the count of one particular genus found divided by the total number of counts of all genera and *s* is the number of genera.

To compare the microbial communities of WTR samples and explore the influence of environment variables and physico-chemical parameters on them, permutational multivariate analysis of variance (PERMANOVA) was conducted on bacterial community results considering the physico-chemical and environmental variables (pH, OM, and available element concentrations) using the Vegan: Community Ecology Package in R. Furthermore, correspondence analysis (CA) and canonical correspondence analysis (CCA) were conducted using the Vegan package to visualise the samples’ bacterial community structures and their associations with environmental variables. Concentrations below the limits of detection were treated as 0 values.

## Results

**Fig. 1:**
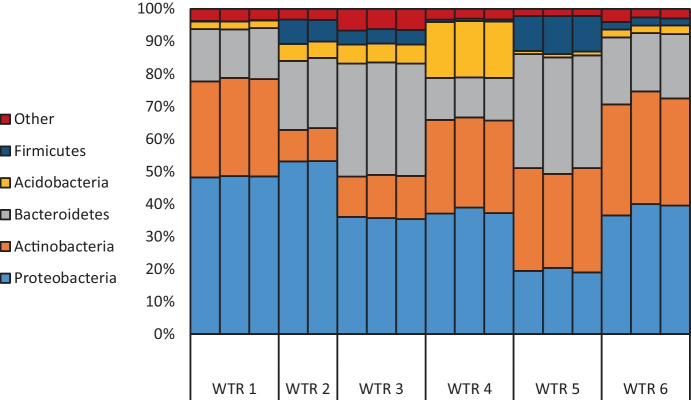
The relative abundance of bacterial communities at phylum level in WTR samples

Following post-analysis processing, at least 33,775 read pairs were obtained per sample of WTRs whilst the negative blank sample produced just 2039 read pairs. The negative blank contained *Actinobacteria*, *Proteobacteria*, *Firmicutes*, and *Bacteroidetes* as the most dominant phyla, accounting for 95% of phyla present in the blank. All of the notable genera and families discussed herein were present in the negative control at under 0.01% relative abundance, aside from *CL500-29* marine group which had a relative abundance of 0.74% in the negative control. This confirms that results obtained for WTR samples directly reflect their microbial community composition and were not affected by any contamination in the laboratory. The reproducibility of samples was good (e.g. Figure 1). Community composition was explored at phylum and genus levels, whilst analysis of taxonomic richness and diversity and component analysis were conducted at genus level.

### Variations in phylum community composition

The most abundant phyla in WTR samples were *Proteobacteria*, *Actinobacteria*, *Bacteroidetes*, *Acidobacteria*, and *Firmicutes* (Fig. 1). Across all WTRs *Proteobacteria*, *Actinobacteria*, and *Bacteroidetes* accounted for 80% or more relative abundance, with *Acidobacteria* accounting for < 10% except in the case of WTR 4 for which it was closer to 20% (Fig. 1).

## Relative abundance of specific genera

The most abundant genera in reservoir-sourced WTRs were *HgcI* clade (also known as acI) and *CL500-29* marine group (11.09 ± 3.78% and 6.77 ± 3.92% relative abundance, respectively, in all reservoir samples; supplementary information Table S1), both of which are members of the Actinobacteria phyla. The reservoir-sourced WTRs were thus rather consistent in terms of most dominant genera aside from sample WTR 4 which was an Fe-based WTR sourced from a reservoir in Wales and for which the abundance of *CL500-29* was lower than in the other reservoir samples and in which, in addition to *HgcI* clade, *Geothrix* and a not yet cultured genus of the *Nitrosomonadaceae* family were prominent genera (8.78 ± 0.20% and 5.03 ± 0.11%, respectively).

Whilst members of the *HgcI* clade were abundant in the reservoir WTRs they were in significantly lower abundance in the river water-sourced WTRs (only 0.94 ± 0.35%; supplementary information Table S1), and members of the *CL500-29* marine group were also at much lower abundances in river-sourced WTRs compared with most of the reservoir WTRs. River-sourced samples (one from England and one from Wales, and also differing in principle coagulant type) were found to be widely different from one another in terms of microbial composition with only a narrow range of common genera that had substantial abundance in both of these WTRs; of those genera, the most abundant were *Geothrix* and *Geobacter* and both of these represented modest overall proportions (each typically representing 1.53 ± 0.59% and 1.26 ± 0.015%, respectively; supplementary information Table S1). Nevertheless, some of these genera common to both of the river-sourced WTRs were at very low abundances or even absent from the reservoir WTRs, including *Geobacter* (an anaerobic bacteria with bioremediation capacity), *Novosphingobium* (also known to degrade a wide range of xenobiotic compounds), *Ruminococcaceae* (found in the healthy human gut), and *Desulfatirhabdium* (an anaerobic, sulphur-reducing bacteria). Conversely, the genus *Candidatus Methylopumilus* (predominantly a freshwater pelagic bacterium) and *Thiobacillus* had orders of magnitude greater abundances in the reservoir samples relative to the river-sourced WTRs (supplementary information Table S1).

Scottish reservoir samples had an abundant presence of *Clostridium *sensu stricto 9 and *Flavobacterium* (5.06 ± 3.17% and 4.97 ± 1.87%, respectively; supplementary information Table S1). Neither of these were found in other samples aside from *Flavobacterium* in WTR 2 (1.64 ± 0.047%) which, possibly coincidently, was also an Al WTR (as were those from Scotland).

**Fig. 2:**
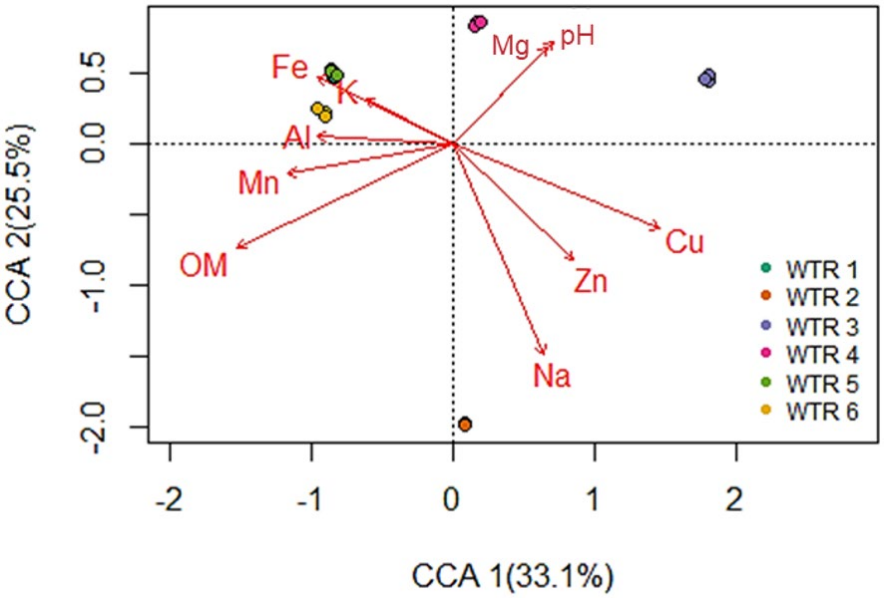
A canonical correspondence analysis of the square root-transformed genus-level microbial community data within WTRs with respect to the measured environmental factors. Arrows indicate the direction and magnitude of a variable’s correspondence. Note that WTR 1 and WTR 5 samples overlap, obscuring WTR 1 symbols in the plot

Correspondence analysis and subsequent canonical correspondence analysis were conducted on square root-transformed WTR sample data to explore the effect of environmental variables on microbial community structure. Reservoir samples (WTR 1, WTR 4, WTR 5, and WTR 6) were generally clustered together whilst river samples (WTR 2 and WTR 3) plotted separately from this cluster (Fig. 2). ANOVA analysis of the CCA revealed that differences in the bacterial community of samples could be partly associated with the examined physico-chemical and environmental variables (*p* < 0.05); the first two components in the CCA explained 58.6% of the variance. However, PERMANOVA analysis of the bacterial counts versus environmental variables showed that none of the environmental variables had a significant influence on bacterial structure when considered on their own (*p* > 0.05).

## Taxonomic richness and diversity

**Fig. 3:**
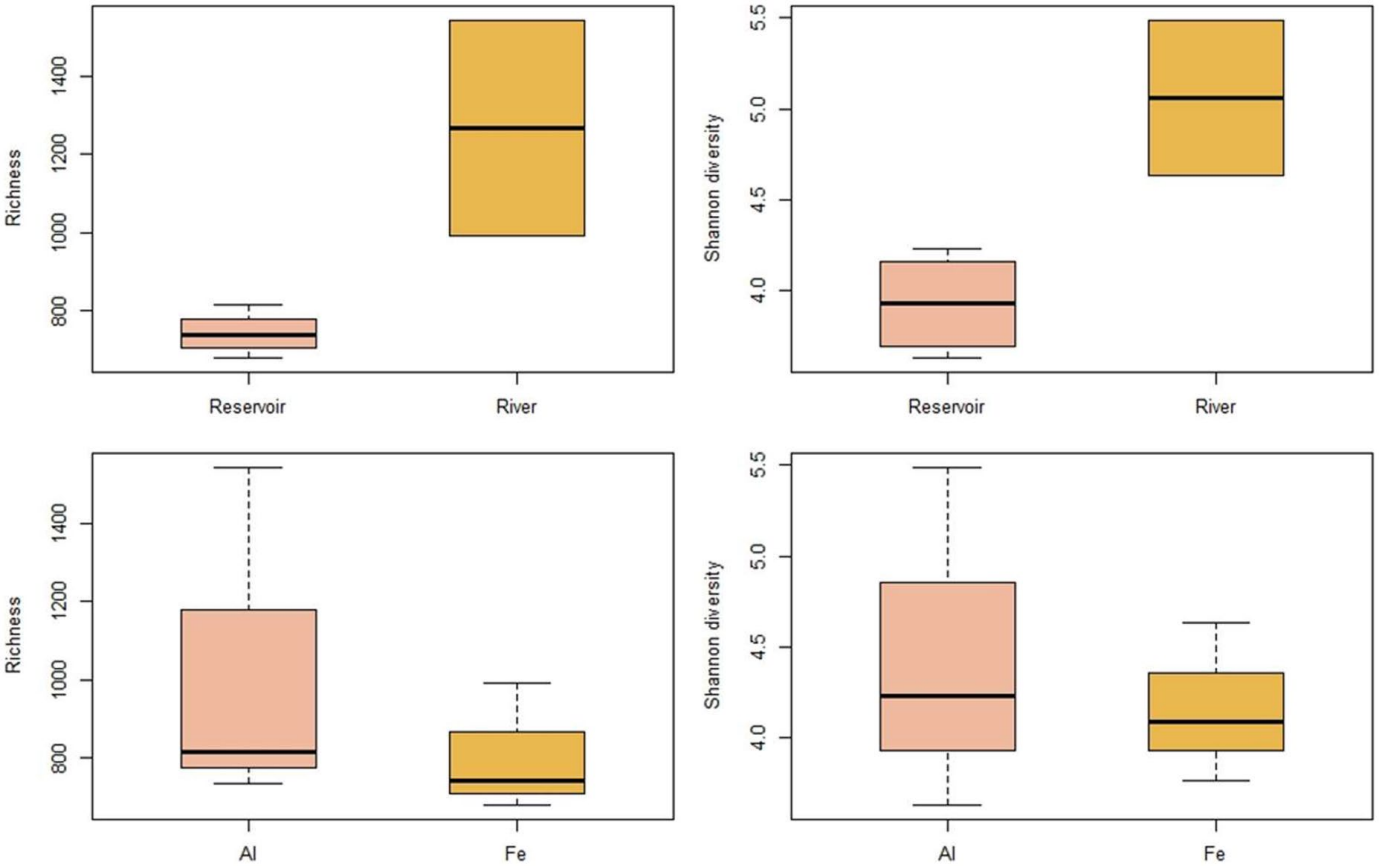
The richness and Shannon diversity index of genera in samples grouped according to differing water sources of WTR samples (*n* = 2 for river water sourced and *n* = 4 for reservoir water sourced) (top panel) and different coagulants used in the production of WTR samples (*n* = 3 for each of Al and Fe salt types) (bottom panel). Boxes indicate median (bold, central line) and inter-quartile range, whilst whiskers indicate 1.5 times the interquartile range where *n* > 2

River-sourced WTR samples had significantly higher average bacterial community diversity than reservoir-sourced samples (Shannon diversity index of 5.06 versus 3.93; ANOVA, *p* < 0.01) and total average genus richness (1269 and 735, respectively; Mann–Whitney *U* test, *p* < 0.01) was also significantly higher (Fig. 3). However, coagulant type did not significantly impact either of these parameters (Mann–Whitney *U* test, *p* > 0.05) (Fig. 3).

## Functional profiling

**Fig. 4:**
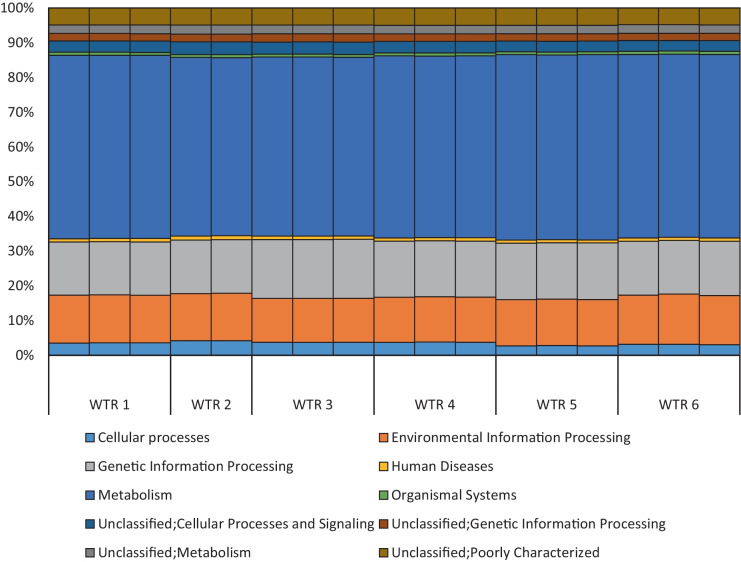
Predicted functional profiling results from PICRUSt analysis of samples. Percentage indicates relative abundance of genomic functional groups categorised according to level 2 groupings of the Kyoto Encyclopaedia of Genes and Genomes (KEGG) Orthology groups (www.genome.jp/kegg)

The results of PICRUSt analysis showed that the genomic functional profiles of the WTRs were all very similar, with the largest proportions of bacterial genome functionality being linked with metabolism, genetic information processing (including transcription, translation, replication, and repair), and environmental information processing (including membrane transport, signal transduction, and related functions) (Fig. 4). This indicates that despite shifts in relative dominance of various phyla and genera amongst the WTRs, their functional gene profiles were essentially equivalent, and therefore, their bacterial functional capacity (i.e. capability of facilitating bacterial mediated processes) would be anticipated to be similar.

## Discussion

### Community composition

At phylum level, *Proteobacteria, Actinobacteria,* and *Bacteroidetes* were consistently the most abundant, which may have positive implications for restoring or enhancing soil microbial functionality if WTRs are added as soil improvers considering that they are also dominant bacterial phyla in soil (Larsbrink & McKee, 2020; Spain et al., 2009), with *Proteobacteria* encompassing a vast diversity of bacteria known to play key roles in carbon, nitrogen, and sulphur cycling (Spain et al., 2009); *Actinobacteria* having been recognised as amongst the most important litter decomposers and thus nutrient-releasing organisms in soil (Kopecky et al., 2011); and *Bacteroidetes* known to be responsible for many extracellular enzyme-catalysed processes in soil (Larsbrink & McKee, 2020). At genus level there is also evidence to suggest that WTR addition may offer potential benefits in terms of restoring or enhancing soil function via contribution of genera known to have capacity for biodegradation of contaminants and for soil bioremediation, e.g. *Geobacter, Novosphingobium, Acinetobacter* (WTR 2), and *Bacillus* (see supplementary information Table S1, Wang et al. (2018), and Anno et al. (2021) for the discussion of biodegradation roles). The relative homogeneity of composition observed amongst subsamples (replicates) of each separate WTR indicates that a microbial community had effectively become established within each and that the sample processing steps (which included thorough mixing by hand before subsampling) were sufficient to ensure that subsamples were representative. Such homogeneity within WTR samples has also been reported previously (Xu et al., 2018), whilst Wang et al. (2021b) found that WTRs that originally differed in microbial community structure became more similar after dewatering or drying (the WTRs in the present study had all been partially dewatered or dried before storage and sampling). To our knowledge, the present study is the first to examine bacterial communities in such previously stored WTRs that have likely developed stable communities, with the handful of previous studies that have been reported on the subject having focussed more on freshly produced WTRs (Ai et al., 2020; Xu et al., 2018).

The predominant phyla in WTR samples analysed in this study were similar to those found by Xu et al. (2018) in WTRs from China; however, that study found a higher relative abundance of *Planctomycetes* and *Cyanobacteria* in some samples (e.g. up to 18% versus 0.039% and up to 49.7% versus 0.37%, respectively) and a lower relative abundance of *Actinobacteria* and *Acidobacteria*. The lower abundance of *Cyanobacteria* in WTRs from the UK found in the present study compared with the Chinese studies may simply reflect cooler temperatures and lower solar irradiance levels in the UK (Solargis, 2020). This is important from a management perspective as many species of *Cyanobacteria* have been found to produce toxins that can pose a risk to human health and are often encountered in freshwater environments (Briand et al., 2003; Falconer, 1996). Of the *Cyanobacteria* found in samples of the present study, none were associated with common potentially toxic bacteria forms. However, whilst differences in raw water parameters and in treatment approaches are likely to be the primary reasons for this, the effects of sampling season differences between the two studies cannot be ruled out as a factor (i.e. February in the present study vs October in that by Xu et al. (2018)); therefore, further seasonal/temporal sampling could be undertaken to explore this.

The two genera found to be dominant in WTRs generated from raw water abstracted from reservoirs, namely the *HgcI* clade and *CL500-29* marine group, match with what is known about their distributions in that they are common and abundant in a wide range of freshwater habitats and particularly in reservoirs and lakes (Ram et al., 2019; Warnecke et al., 2004). *HgcI* genera are associated with nutrient cycling (Ghylin et al., 2014), whereas members of the *CL500-29* clade are known to be generalist that can utilise different carbon sources (Lindh et al., 2015). Their greater relative abundance in reservoir samples may be due to longer water residence times and subsequent increased nutrient cycling levels in reservoirs relative to rivers.

The two river water-sourced WTR samples (WTR 2 and WTR 3) differed from one another in microbial composition, which could be due to differences in the original raw water linked to their different geographical locations. In these, and other WTRs, the presence of strictly anaerobic genera such as *Geobacter* and *Geothrix* that are found in Fe-reducing environments (Coates et al., 1999; Lovley et al., 2011) suggests that anaerobic microsites existed at some stage during WTR formation and storage. *Geobacter* species reportedly may also play a role in the reduction of As and increase its mobility from sediments (Wang et al., 2019).

The *Flavobacterium* genus which was present in Al-based WTRs is commonly found in most aquatic ecosystems varying from freshwater to saline, and plays a role in denitrification (Boone et al., 2001; Schaechter, 2009). The lack of other highly abundant genera being significantly affected by WTR coagulant type is likely due to coagulant type having little influence on important parameters such as available element concentrations and organic matter content. For example, WTR 1, which is Fe based, had similar concentrations of available Al as WTR 2 which is Al based. Similarly, WTR 3 had no detectable Fe in its leachate although it is Fe salt based.

*Clostridium *sensu stricto 9 which was abundant in only the WTR samples from Scotland (both of which were reservoir based and of the Al-WTR type) may be important as the *Clostridium* genus, whilst also containing many commonly occurring and harmless free-living bacteria, contains species that are harmful to human and animal health including *C. botulinum* and *C. tetan* which are causative of botulism and tetanus, respectively (Schaechter, 2009). Therefore, further examination of the *Clostridium* to a species level for any potential negative associations is warranted.

## Functional profiling

The dominant genomic functionalities identified from PICRUSt analysis of the DNA data were principally related to cell function and structure maintenance and to reproduction; these are key functions undertaken by all species present. The similar functional profiles between the various WTR samples may be an example of functional redundancy within a microbial community, as the same functional roles can be fulfilled by multiple species. For example, both *Bacillus* and *Pseudomonas* are known for their generation of lipases and other important enzymes that break down lipids in organic matter (Bharathi & Rajalakshmi, 2019), meaning that the presence of either genus can ensure that this function is fulfilled.

Functional redundancy is present in soils, and therefore may also be present in WTRs due to their many physical and chemical similarities (Grządziel, 2017). In a study of bacterial communities in soils from areas with varying land usages, Sengupta et al. (2020) found genomic functionality to be dominated by similar types of functions as those observed for WTRs in the present study. Moreover, we found high genomic functionality similarities between bacterial communities in the WTRs and a reference soil tested in our laboratory (detailed in Figure S1 in the Supporting information), providing further indication that bacterial communities in WTRs and soils are comparable and compatible in terms of composition and function. Of course, functions driven or enhanced by fungi in soil, and how fungal communities may be disturbed or influenced by WTR addition, has not been addressed by the bacterial investigation conducted here. Considering the importance of fungi to soil health (e.g. Frąc et al., 2018), the fungal diversity and composition of fungal communities in WTRs warrant similar investigation.

## Conclusions

Analysis of sequenced DNA of WTRs from the UK revealed that the predominant phyla of bacteria and associated microbes were similar to those found in studies of WTRs from China, showing some level of universality. However, important differences in cyanobacteria and pathogenic microbe abundance were identified that can be linked to water quality, treatment processes, and climatic parameters. Based on these results, it is unlikely that the WTRs from the UK investigated in this study would negatively impact soil microbial functions when utilised for agricultural land application but may in fact offer some potential enhancement or restoration. Whilst the specific microbial composition of WTRs samples varied, the genomic functionality profiles were similar to each other and to that reported for soils, further indicating compatibility for WTR deployment in the landscape.

Two factors that are known to control the chemical composition of WTRs, water source and coagulant use, were explored as controlling variables on microbial composition. River-sourced WTR samples had significantly higher bacterial community diversity and genus richness than reservoir samples; however, coagulant type did not play a significant role in diversity and richness. Furthermore, canonical correspondence analysis of microbial composition in WTRs clearly separated samples based on their water source type (river, reservoir), but not principal treatment salt used in their production (Al or Fe). Additionally, overall, the physical and environmental variables explored in this study could, when combined together, partly predict the microbial structure of WTRs but no individual controlling variable had a significant effect on WTR bacterial population composition. The presence of *Geobacter* and *Geothrix* within river samples is indicative of Fe-reducing anaerobic environments. Other avenues of research warranting further investigation are microbial differences in WTRs generated in different seasons, and fungal diversity in WTRs and implications regarding compatibility with soil microflora.

### Supplementary Information

Below is the link to the electronic supplementary material.

Supplementary file1 (DOCX 133 KB)

Supplementary file2 (XLSX 165 KB)

## Data Availability

Any data beyond that contained in the article and the supporting information can be obtained from the corresponding author upon reasonable request.
