# Supplementary material for: Microbial genome (Illumina MiSeq) sequencing of drinking water treatment residuals to evaluate compatibility with environmental applications
Source: Environ Monit Assess. 2023 Aug 9;195(9):1027. doi: 10.1007/s10661-023-11511-3 (PMC10409814; doi:10.1007/s10661-023-11511-3)
Supplement: Supplementary file 1 — Supplementary file1 (DOCX 133 KB) [file 10661_2023_11511_MOESM1_ESM.docx]

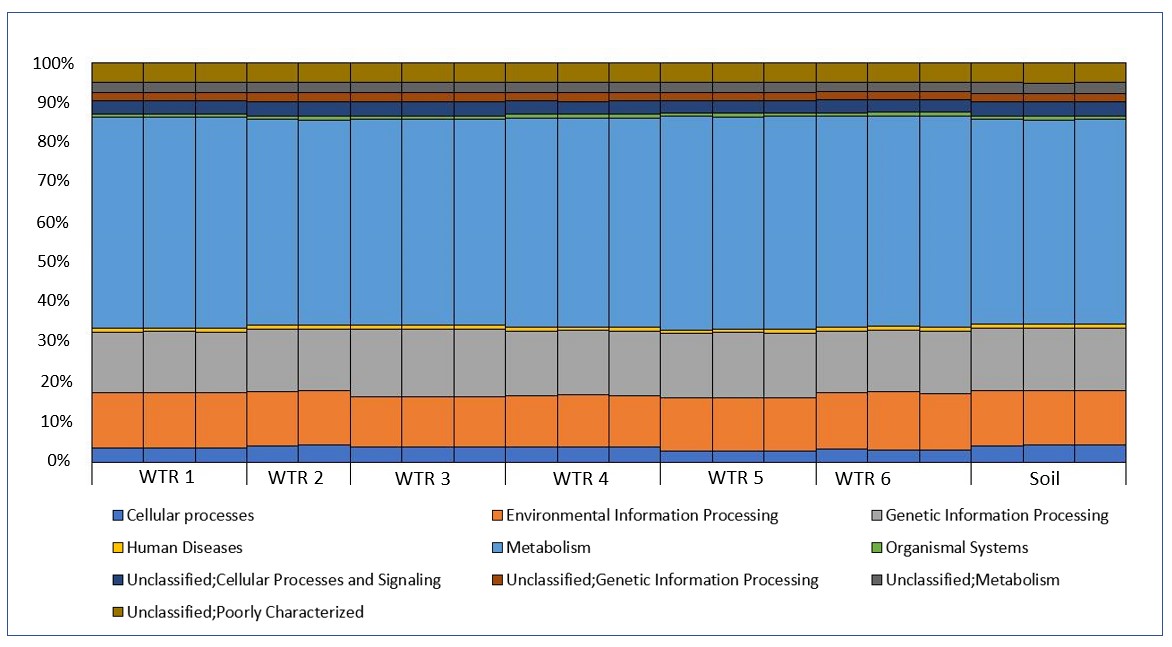
Figure S1

Supplementary Information Figure S1. Predicted functional profiling results from PICRUSt analysis of DNA sequencing of WTR samples and a sample of reference soil from Wales (the soil had not been treated with WTRs but land elsewhere in the local region does receive periodic WTR applications). Percentages indicate relative abundance of genomic functional groups categorised according to level 2 groupings of the Kyoto Encyclopaedia of Genes and Genomes (KEGG) by KEGG Orthology groups (www.genome.jp/kegg).
